# Prevalidation of an ELISA for Detection of a New Clinical Entity: *Leishmania donovani*-Induced Cutaneous Leishmaniasis

**DOI:** 10.1155/2020/9289651

**Published:** 2020-07-15

**Authors:** Bhagya Deepachandi, Sudath Weerasinghe, Himali Gunathilake, Thisira P. Andrahennadi, Mahendra N. Wickramanayake, Shantha Siri, Vishvanath Chandrasekharan, Preethi Soysa, Yamuna Siriwardana

**Affiliations:** ^1^Department of Parasitology, Faculty of Medicine, University of Colombo, Colombo 00800, Sri Lanka; ^2^Department of Biochemistry and Molecular Biology, Faculty of Medicine, University of Colombo, Colombo 00800, Sri Lanka; ^3^Department of Chemistry, Faculty of Science, University of Colombo, Colombo 00300, Sri Lanka; ^4^National Science Foundation, 47/5 Maitland Place, Colombo 00700, Sri Lanka

## Abstract

Human leishmaniasis which is considered a neglected tropical parasitic disease presents in three main clinical forms (i.e., cutaneous leishmaniasis (CL), mucocutaneous leishmaniasis (MCL), and visceral leishmaniasis (VL)) that are mainly determined by its causative species. *Leishmania donovani*, the most virulent and visceralizing parasite, is increasingly reported to cause CL in many countries in the world. Although CL is generally not considered to evoke a humoral immune response except for a nonrobust and a variable response in minority of cases, VL is associated with a clear strong humoral response. However, humoral response in *L*. *donovani*-induced CL has not been well evaluated before. A suitable serology-based assay is an essential primary step in such a study. An indirect enzyme-linked immunosorbent assay (ELISA) based on *Leishmania* promastigote crude antigen (Ag) was designed and optimized in order to utilize in further serological studies on this new clinical entity. Optimization included quantification of crude Ag, checkerboard titration method for determination of optimal concentrations for coating Ag, human sera and secondary antibody (Ab) with suitable coating buffer, blocking buffer, and incubating temperatures. The selected coating buffer was 0.02 M phosphate buffer, pH 6.8, and the blocking buffer was 2% fetal bovine serum with 0.01 M phosphate-buffered saline. At least 1 *μ*g of crude Ag was required for coating the ELISA plate, while 1 : 1000 serum was used as primary Ab. The optimized concentration of secondary Ab was 1 : 64000 which might be altered according to manufacturer recommendations. The assay specificity was pre-evaluated using sera (*n* = 20 from each category) from confirmed CL patients and controls (other skin diseases which mimic CL, other systemic diseases that mimic VL, nonendemic healthy controls, and endemic healthy controls). This procedure described an optimization procedure of an ELISA technique for detection of anti-*Leishmania* antibodies in patients with *L*. *donovani* caused CL.

## 1. Introduction

Leishmaniasis, a parasitic disease found in parts of the tropics, subtropics, and Southern Europe present as one of the three clinical forms: cutaneous leishmaniasis (CL), visceral leishmaniasis (VL), and mucocutaneous leishmaniasis (MCL) which are mainly species dependant. Untreated VL and MCL potentially fatal while CL is associated with great morbidity [1]. CL induced by *L*. *donovani* which is the known cause of VL is increasingly reported at a global scale [2–4]. Humoral response against the mismatching clinical and parasite scenario has not been examined so far [5].

Sri Lanka sets an ideal example in the southasian region where more than 6000 cases of CL have been notified during past 10 years [6]. *L*. *donovani* was identified as the causative agent of CL in this setting [7–10]. Main clinical entity remained as CL in a clear majority of reported cases over the time [11–16].

Skin and mucosal infections result mainly in cell-mediated immunity, while visceralizing parasites exert a humoral response [17]. This has enabled development and effective utilization of many serological tools for diagnosis of VL [18–22]. Usefulness of serology in diagnosis of CL has shown to be less useful with highly variable and usually weak response [23, 24].

However, owing to the unexamined visceralizing potential of the local causative species, examination of the serological response was thought to be useful in this clinical entity. Further confirming this, two preliminary studies reported a seroprevalence among local CL during the first attempt [5, 25]. The seroprevalence was initially found as 14.0% [25]. Subsequently, a seroprevalence of 34.0% was reported with modifications to the technique indicating the possibility for further improvement [5]. However, specificity of the tool was not examined during these two attempts. Meanwhile, changing trends in CL profile with different disease transmission foci, atypical CL forms, and emergence of VL and MCL have also been reported in the country, justifying the further examination of serological aspects of *L*. *donovani*-induced CL [16, 26–30].

Accurate determination of the actual seroprevalence also enables decision-making with regard to the usefulness of its further development as a diagnostic or a research tool. The aim of this study was to further optimize and examine the specificity of the previously established ELISA technique with a view to revisit the seroprevalence of *L*. *donovani*-induced CL.

## 2. Materials and Methods

### 2.1. Setting and Timing

This study was carried out in Sri Lanka on locally acquired cases of CL reported in local patients diagnosed during 2014–2016.

### 2.2. Instrumentation, Materials, and Reagents

Absorbance measurements were obtained by the Thermo Electron Corporation Multiskan EX microplate reader. Micropipettes (0–20 *μ*l, 20–200 *μ*l, and 100–1000 *μ*l Nichipet EXII micropipettes from Nichiryo) and microwell plates (96 wells) (Sterilin, Tentorio, Italy) were used. Horseradish peroxidase conjugate-goat anti-human immunoglobulin (gamma chain) secondary Ab (goat anti-human IgG-HRP) and 3,3′,5,5-tetramethylbenzidine (TMB) chromogen substrate solution were purchased from Invitrogen (Camarillo, California). Penicillin-streptomycin (Penstrep), heat-inactivated fetal bovine serum (HI-FBS) and medium 199, Hank's balanced salts (M199), and the reagents required for cell culturing were purchased from Gibco (Life Technologies, Grand Island, United States of America). All other chemicals and reagents including, sodium phosphate dibasic (Na_2_HPO_4_), sodium phosphate monobasic (NaH_2_PO_4_), sodium chloride (NaCl), sodium bicarbonate (NaHCO_3_) potassium chloride (KCl), potassium phosphate monobasic (KH_2_PO_4_), sodium carbonate (Na_2_CO_3_), copper sulfate (CuSO_4_), potassium sodium tartrate (KNaC_4_H_4_O_6_), sodium hydroxide (NaOH), Folin and Ciocalteu's phenol reagent, bovine serum albumin (BSA/fraction V), polyethylene glycol sorbitan monolaurate (Tween-20), and sulfuric acid (H_2_SO_4_) were purchased from Sigma-Aldrich (now known as Merck, Saint Louis, Missouri, USA).

### 2.3. Patient Isolates and Preparation of Crude Ag

Clinically suspected patients (*n* = 35) with locally acquired CL were recruited after informed written consent [14]. Lesion aspirates or slit skin scrapings were collected. Diagnosis of CL was confirmed/excluded using light microscopy (LM), in vitro culturing (IVC), and/or polymerase chain reaction (PCR) [31–33].

Clinical isolates were used to inoculate complete medium 199 (M199) supplemented with 20% HI-FBS and 0.1% Penstrep [34]. Parasites at the late log phase with an average density of 1 × 10^7^ cells/ml were harvested [34]. Crude Ag was extracted from the harvested promastigotes of *Leishmania* using the freeze-thawing method [35]. The pellet was washed four times in cold 0.01 M phosphate-buffered saline (PBS), pH 7.4, and resuspended at a concentration of 1.0 g of cell pellet in 2 ml of cold 0.01 M PBS, pH 7.4. Subsequently, the suspension was freeze-thawed (freezing for 30 seconds in liquid nitrogen and thawing at room temperature) for three times. The suspension contained the total crude Ag, and it was aliquoted and stored at −20°C.

### 2.4. Protein Estimation

Extracted crude Ag was quantified using a modified Lowry assay which was developed and validated within home laboratory settings [36]. Briefly, BSA was used as the standards (100–500 *μ*g/*μ*l). Standards or the crude Ag sample (100 *μ*l) was added to separate wells and mixed with 20 *μ*l of NaOH (2 N) in a plate shaker for 10 minutes. A volume of 100 *μ*l of reagent mixture A (2% Na_2_CO_3_, 1% CuSO_4_ and 2% KNaC_4_H_4_O_6_ in 100 : 1 : 1 ratio) was added to each well and mixed well for 5 minutes followed by incubation at room temperature for 10 minutes. Folin and Ciocalteu's phenol reagent (2 N, 20 *μ*l) was added and mixed well immediately and incubated at room temperature in dark conditions for 30 minutes. Absorbance was read at 650 nm using an ELISA reader.

### 2.5. Indirect ELISA

A ninety-six-well ELISA plate (Sterilin, Tentorio, Italia) was coated with 100 *μ*l of extracted crude protein (containing at least 1 *μ*g protein) dissolved in 0.02 M phosphate buffer, pH 6.8, and incubated over night at 4°C [21, 22]. Thereafter, the coated wells were washed 3 times with 0.01 M phosphate-buffered saline (PBS) with 0.1% tween-20 (PBST) and incubated with 200 *μ*l of blocking buffer (0.01 M PBS with 2% FBS) at room temperature for 6 to 8 hours. Reference Abs (sera) at appropriate dilutions were added and incubated overnight at 4°C [21, 22]. Following overnight incubation, the plate was washed with PBST for 3 times and 100 *μ*l goat anti-human IgG-HRP (at appropriate dilution) was added to each well. After 30 minutes at 37°C, the plate was washed with PBST for 6 times with 5 minutes gentle shaking for last 5 washings. Subsequently, the plate was incubated with 100 *μ*l of TMB (tetramethyl benzidine) substrate solution at room temperature for 30 minutes, and substrate reaction was stopped by adding 100 *μ*l of 1 N H_2_SO_4_. Absorbance values were read at 450 nm using an ELISA reader [21, 22].

### 2.6. Checkerboard Titration (CBT) of ELISA

**Figure 1:**
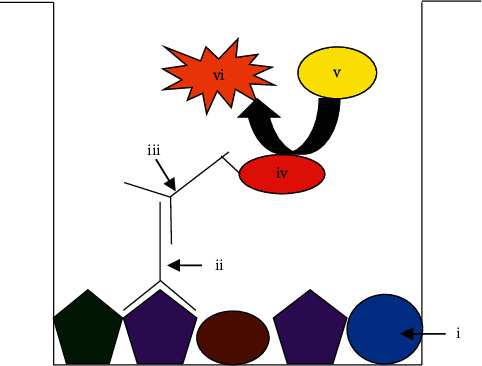
Schematic representation of indirect ELISA method used for the study. (i) Crude *Leishmania* Ag, (ii) anti-*Leishmania* IgG Ab in human serum, (iii) peroxidase-goat anti-human IgG secondary Ab, (iv) HRP, (v) TMB substrate, and (vi) oxidized TMB.

Concentration of reagents required for indirect ELISA (Figure 1) including crude Ag, human serum (primary Ab), and goat anti-human IgG-HRP (secondary Ab) was determined using the CBT method described by Crowther [37]. Initially, crude Ag was diluted rowwise on the ELISA plate with known amounts of protein [37]. Preliminary studies were carried out (data are not shown) to determine the best range of Ag (0.5–2.5 *μ*g) for coating the ELISA plate. Only diluent (PBS) was added to the last row. Sera were diluted columnwise (preliminary studies were carried out to determine the best range of serum dilution (1 : 500–1 : 5000); data are not shown), and only diluent (PBS-FBS) was added to the last column [37]. Anti-species conjugate was added at single dilution (1 : 4000 is the recommended dilution from the commercial supplier). The best Ag dilution was estimated where there was an adequate color intensity as a result of binding with antibodies [37].

Using the optimized amount of coating Ag, the optimized dilutions of primary and secondary Ab were determined. The plate was coated with optimized amount of coating Ag, and the dilution range of the serum was added as described. The serum was diluted columnwise on the ELISA plate starting with the highest concentration, while anti-species conjugate was diluted rowwise on the ELISA plate (preliminary studies were carried out to determine the best dilution range of anti-species conjugate (1 : 32000–1 : 64000); data are not shown). The best dilutions at the point of a high binding ratio (BR) with a high assay sensitivity were selected as the optimized dilutions of two reagents.

### 2.7. Serum Collection, Preparation, and Specificity Assay

Serum samples from confirmed locally acquired CL (*n* = 20) cases and equal numbers (*n* = 20) from each group of controls including NCL, NVL, NEHC, and EHC were used for the study. If positive patients for CL were with a history of overseas travel within two years prior to diagnosis, they were excluded from the study. NCL samples were collected from patients admitted to Dermatology Ward at National Hospital of Sri Lanka (NHSL) which included patients with infectious skin conditions (leprosy and cutaneous tuberculosis (*n* = 8)) and immunological conditions (contact dermatitis, eczema, and psoriasis (*n* = 12)). NVL samples were collected from patients with other systemic infectious diseases (dengue, leptospirosis, pyrexia of unknown origin, and hepatomegaly with/or splenomegaly (*n* = 13)) and immunological disorder (carcinoma and systemic lupus erythematosus (*n* = 7)). Sera were collected from the healthy persons who lived in the western province which is not considered as a leishmanial area (*n* = 20) and healthy persons who lived in Southern Province, a disease-endemic area (*n* = 20).

Laboratory confirmation of samples was done by LM, IVC, and/or PCR [31–33]. Venous blood (approximately about 3 cc) was collected from each participant to plain blood tubes. They were incubated at room temperature for 30 minutes to 1 hour to allow for blood clotting. Sera were separated by centrifugation at 2500 rpm for 10–15 minutes, aliquoted, and stored at −20°C for later use.

Optimized conditions were applied for pre-evaluation of the assay for specific detection of leishmaniasis. Unless otherwise stated, a minimum of two replicates were used for determining mean optical density values of samples in quantification of ELISA absorbance values.

## 3. Results

**Figure 2:**
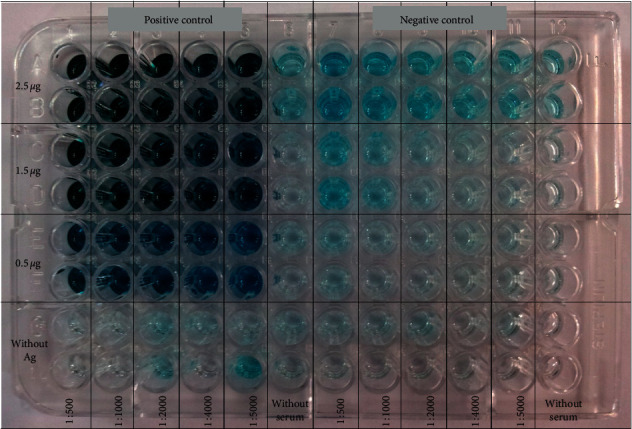
CBT of crude Ag concentration against serum after 30 minutes from adding TMB substrate solution. Ag was diluted rowwise, and positive and negative sera were diluted columnwise. Duplicates were done for each condition.

According to the modified Lowry assay method, the yield of the crude Ag extract was 15 mg for 1 g of the parasite cell pellet [36]. Selected amounts of crude Ag (0.5 *μ*g, 1.5 *μ*g, and 2.5 *μ*g) were initially used for coating the microwell plate, and the outcome of ELISA was checked. Five dilutions of primary Ab (1 : 500, 1 : 1000, 1 : 2000, 1 : 4000, and 1 : 5000) were used (Figure 2). Results obtained for a positive serum was compared with the results obtained for a healthy control, and BRs were calculated. A higher BR was observed in 0.5 *μ*g and 1.5 *μ*g than in 2.5 *μ*g. Therefore, the same experiment was repeated with 0.5 *μ*g, 1.0 *μ*g, and 1.5 *μ*g of crude Ag. Since the values were almost similar for all three concentrations, the middle value, i.e., 1.0 *μ*g, was selected as the best.

**Figure 3:**
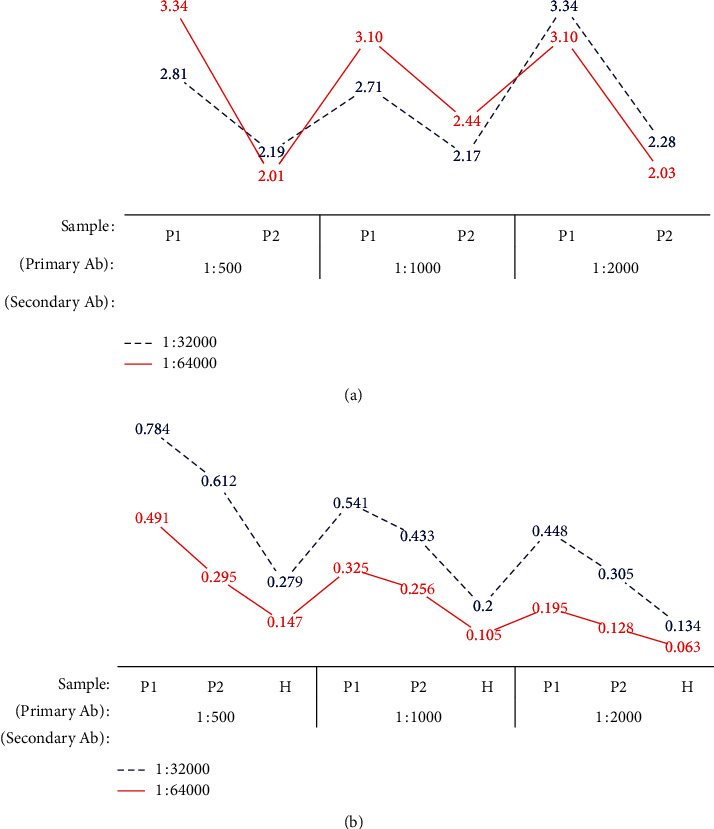
The variation of BR and ELISA absorbance values in different concentrations of primary and secondary Ab concentration (P1: high positive serum, P2: low positive serum, and H: healthy serum). (a) Variation of BR. (b) Variation of ELISA absorbance values.

Using the optimized quantities of crude Ag (1.0 *μ*g/well), primary Ab concentration was optimized as described. BR was increased with the concentration of primary antibodies while secondary antibodies were decreased (Figure 3). According to the data obtained for ELISA absorbance values, 1 : 1000 and 1 : 64000 were selected as the best dilutions for serum (primary Ab) and anti-species conjugate (secondary Ab), respectively, where both BR and absorbance values were high.

**Figure 4:**
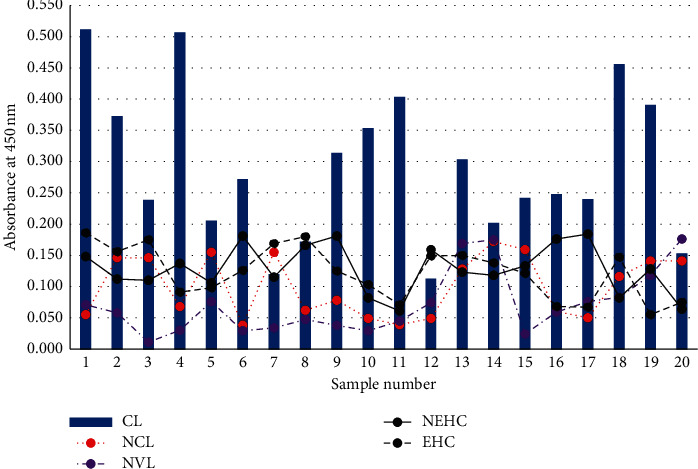
ELISA absorbance values of CL positives and other control samples. Twenty different samples from each group were used for comparison.

In the pre-evaluation of specificity of the optimized ELISA method, high absorbance values were observed with CL sera compared with control samples except 4 out of 20 CL sera which showed comparably lower absorbance values than other CL sera (Figure 4).

## 4. Discussion

In this study, we further optimized and examined an in-house ELISA which is important to evaluate the humoral response in leishmaniasis in settings with *L*. *donovani*-induced CL.

Among different methods of ELISA, the indirect method was selected as it was user-friendly and most suitable for the requirement compared with other ELISA methods [37]. The indirect ELISA method was developed for diagnosis of leishmaniasis in disease-endemic areas in other countries [21, 38, 39]. Because of its wide usage in diagnosis, the same technique optimized using local parasite Ag was applied on local CL cases.

In CBT, concentrations of two reagents were altered columnwise and rowwise on the microwell plate (Figure 2) at the same time. The concentrations at the best point which had high assay sensitivity were selected as the optimized concentrations for the two reagents [37]. Using the CBT method, the optimal concentrations for crude Ag, patient sera, and anti-species conjugate were optimized as 1 *μ*g, 1 : 1000, and 1 : 64000, respectively. The dilutions of human sera used for ELISA are usually varied from 1 : 500 to 1 : 1000 [21, 22]. Therefore, 1 : 1000 was reasonable, and it may have high amount of disease specific Abs than using higher dilutions at more than 1 : 1000. The dilution of a secondary Ab was mainly dependent on the initial concentration of the stock solution, sample source, and disease under examination.

BR was the ratio between respective absorbance values obtained for the positive control and the healthy control [37]. We were able to overcome the errors occurred due to nonspecific binding in healthy controls using BR when analyzing the ELISA results. High concentrations of secondary Ab may cause a reduction in the BR by increasing the nonspecific amplifications in healthy controls. P1, P2, and H were analyzed and compared for the BR, and absorbance values are depicted in Figure 3. At the lower dilutions of secondary Ab, the BR of P2 was reduced due to increased nonspecific bindings. Also, in the higher dilutions of secondary Ab, because of the reduction of absorbance values, the BR ratio was reduced. The comparison of both BR and absorbance values of ELISA together was important to select the optimum dilutions for primary and secondary Abs.

We successfully applied the optimized method for a study group which consisted of *n* = 20 sera from each category of CL, NCL, NVL, NEHC, and EHC to analyze the binding specificity of the assay. Out of the study group, *n* = 16/20 CL (80%) patients gave remarkably high absorbance for ELISA than healthy control sera (Figure 4) which could be useful for discrimination of seropositive cases from healthy individuals.

## 5. Conclusions

In the current study, we described crude Ag-based indirect ELISA through altering the concentration of reagents and changing several conditions which would be applicable for other ELISA systems also. This assay may be useful for detection of anti-*Leishmania* IgG in CL caused by *L*. *donovani*. The assay may be evaluated using a larger number of samples, in multiple settings, and for different clinical scenarios in leishmaniasis. Further studies are underway.

Applications were submitted for patenting at the National Intellectual Property Office of Sri Lanka (national patent LK/P/1/19697).

## Data Availability

The data supporting the conclusions of this article are included within the article. Other data have not been made available as they were not part of the ethics application and due to patient confidentiality.
